# Clinical and Molecular Characterization of Patients with Fructose 1,6-Bisphosphatase Deficiency

**DOI:** 10.3390/ijms18040857

**Published:** 2017-04-18

**Authors:** Niu Li, Guoying Chang, Yufei Xu, Yu Ding, Guoqiang Li, Tingting Yu, Yanrong Qing, Juan Li, Yiping Shen, Jian Wang, Xiumin Wang

**Affiliations:** 1 Department of Medical Genetics and Molecular Diagnostic Laboratory, Shanghai Children’s Medical Center, Shanghai Jiaotong University School of Medicine, Shanghai 200127, China; liniu0509@163.com (N.L.); xuyufei82@sjtu.edu.cn (Y.X.); 18817557506@126.com (G.L.); ytt.007@163.com (T.Y.); qingyanrong521@163.com (Y.Q.); yiping.shen@childrens.harvard.edu (Y.S.); 2 Department of Endocrinology and Metabolism, Shanghai Children’s Medical Center, Shanghai Jiaotong University School of Medicine, Shanghai 200127, China; changguoying@126.com (G.C.); dingyu@scmc.com.cn (Y.D.); lijuan@scmc.com.cn (J.L.); 3 Department of Laboratory Medicine, Boston Children’s Hospital, Boston, MA 02115, USA

**Keywords:** fructose-1,6-bisphosphatase (FBPase) deficiency, targeted-next generation sequencing, *FBP1* gene, functional study

## Abstract

Fructose-1,6-bisphosphatase (FBPase) deficiency is a rare, autosomal recessive inherited disease caused by the mutation of the *FBP1* gene, the incidence is estimated to be between 1/350,000 and 1/900,000. The symptoms of affected individuals are non-specific and are easily confused with other metabolic disorders. The present study describes the clinical features of four Chinese pediatric patients who presented with hypoglycemia, hyperlactacidemia, metabolic acidosis, and hyperuricemia. Targeted-next generation sequencing using the Agilent SureSelect XT Inherited Disease Panel was used to screen for causal variants in the genome, and the clinically-relevant variants were subsequently verified using Sanger sequencing. Here, DNA sequencing identified six variations of the *FBP1* gene (NM_000507.3) in the four patients. In Case 1, we found a compound heterozygous mutations of c.704delC (p.Pro235GlnfsX42) (novel) and c.960_961insG (p.Ser321Valfs) (known pathogenic). In Case 2, we found a compound heterozygous mutations of c.825 + 1G>A and c.960_961insG (both were known pathogenically). In Case 3, a homozygous missense mutation of c.355G>A (p.Asp119Asn) (reported in ClinVar database without functional study) was found. Case 4 had a compound heterozygous mutations c.720_729del (p.Tyr241GlyfsX33) (novel) and c.490G>A (p.Gly164Ser) (known pathogenically). Further in vitro studies in the COS-7cell line demonstrated that the mutation of ASP119ASN had no impact on protein expression, but decreased the enzyme activity, and with which the clinical significance of Asp119Asn can be determined to be likely pathogenic. This report not only expands upon the known spectrum of variation of the *FBP1* gene, but also deepens our understanding of the clinical features of FBPase deficiency.

## 1. Introduction

Fructose-1,6-bisphosphatase (FBPase) is one of the key enzymes of gluconeogenesis that catalyzes the splitting of fructose-1,6-bisphosphate (FBP) into fructose 6-phosphate and inorganic phosphate [1]. The FBPase enzyme is mainly expressed in the liver and kidney, two distinct genes, *FBP1* and *FBP2*, which encode human FBPase [2]. The *FBP1* gene (OMIM#611570), which is mainly expressed in liver tissues, is located on 9q22.3 and consists of seven exons that encode 338 amino acids [3]. The *FBP2* gene (OMIM#603027) was initially isolated from muscle tissues and encodes a 339–amino acid protein that shares a 77% amino acid sequence identity with the FBP1 protein [4] (Figure S1, see Supplementary Material).

Mutations in the *FBP1* gene cause FBPase deficiency (OMIM#229700), which is characterized by an impairment of glucose synthesis from all gluconeogenic precursors. It is an autosomal recessive inherited disorder with clinical symptoms of hypoglycemia, ketosis, acidosis, hyperlactacidemia, convulsions, and coma [1]. The non-specific clinical features of FBPase deficiency are easily confused with several other metabolic disorders, such as glucose-6-phosphatase (G6P) deficiency and phosphoglucomutase 1 (PGM1) deficiency, especially in the absence of definitive biochemical findings [5,6]. Measurement of FBPase activity using liver biopsies or leukocytes is apparently the most reliable diagnostic approach to date, although its sensitivity remains relatively low and is generally considered as an invasive medical procedure [7,8]. In addition, the disorder may become fatal when patients develop severe hypoglycemia when glycogen reserves are insufficient (in newborns or fasting individuals) or after a fructose load [9]. Thus, the establishment of a rapid and accurate diagnostic method for FBPase deficiency is imperative.

We hereby report the clinical features and the results of genetic testing of four patients strongly suspected of having inherited metabolic diseases. Using targeted next-generation sequencing (NGS) with the Agilent SureSelect XT Inherited Disease Panel (Agilent Technologies, Inc., Santa Clara, CA, USA), all of the patients were confirmed to harbor pathogenic variants in the *FBP1* gene (NM_000507.3) and were diagnosed with FBPase deficiency. The functions of two variants (Asp119Asn and 704delC) were also evaluated by in vitro experimentation.

## 2. Results

### 2.1. Clinical Description

**Figure 1:**
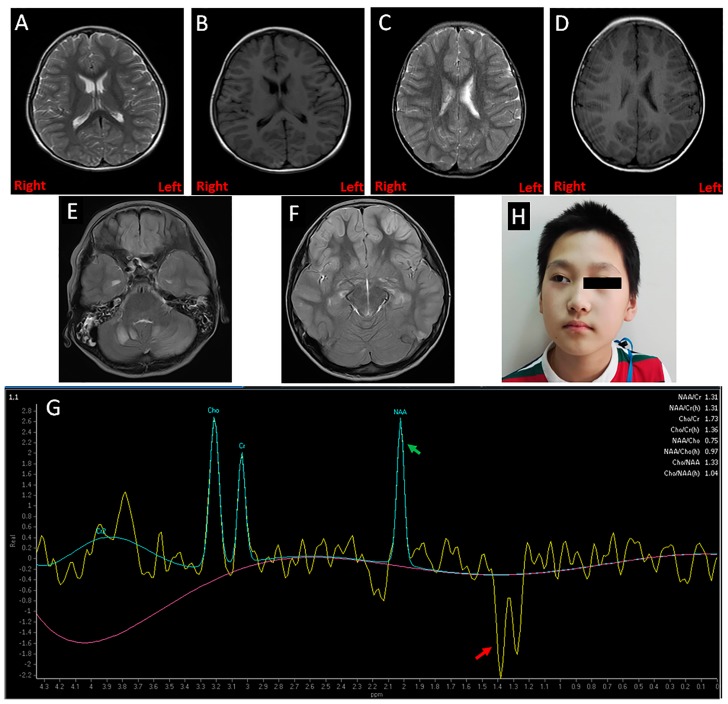
Clinical features of the patients. (**A**,**B**) Magnetic resonance imaging (MRI) showed a mildly enlarged bilateral ventricle in Case 1; (**C**,**D**) A mildly enlarged left ventricle was found in Case 2 by MRI; (**E**,**F**) Several parts of the brain (cerebellum, frontoparietal, basal ganglia, and thalamus) in Case 4 had symmetrical patchy abnormal signals; (**G**) Magnetic resonance spectroscopy (MRS) revealed the brain of Case 4 had an abnormal increased lactate peak and reduced *N*-acetyl aspartate (NAA) peak, which is marked by the red and green arrows, respectively; (**H**) The facial picture of Case 4 with a strabismic right eye.

Case 1 was a girl of five years and six months old. She was referred to the gastroenterology clinic of Shanghai Children’s Medical Center (SCMC) for the chief complaint of fever, vomiting, convulsions, and coma. She was the first child of healthy, non-consanguineous parents and was born at term via normal delivery. When she was 3 days old, she experienced vomiting and was diagnosed with hypoglycemia, hemorrhage of the digestive tract, and metabolic acidosis in a local hospital. Since she was two years old, the patient had been hospitalized several times in local hospitals for these conditions. However, her developmental milestones were normal. Additionally, her two-year-old brother exhibited similar syndromes when he stopped breastfeeding at five months. The laboratory findings of this first patient indicated hypoglycemia (glucose: 0.3 mmol/L), hyperlactacidemia (11.9 mmol/L), metabolic acidosis (pH: 6.97, base excess: −25.0 mmol/L), and hyperuricemia (uric acid: 875 μmol/L). Blood samples obtained during hypoglycemia showed insulin: 8.0 μIU/mL, adrenocorticotropic hormone (ACTH) > 1250 pg/mL, cortisol: 57.3 μg/dL. The electroencephalogram and ultrasound results were normal. The cranial magnetic resonance imaging (MRI) results showed the first patient to have a mild enlarged bilateral ventricle (Figure 1A,B). Ultrasound results of the abdomen, thyroid, and heart were normal. The patient was treated for her symptoms with an infusion of antibiotics, glucose, and bicarbonate, immediately.

**Figure 2:**
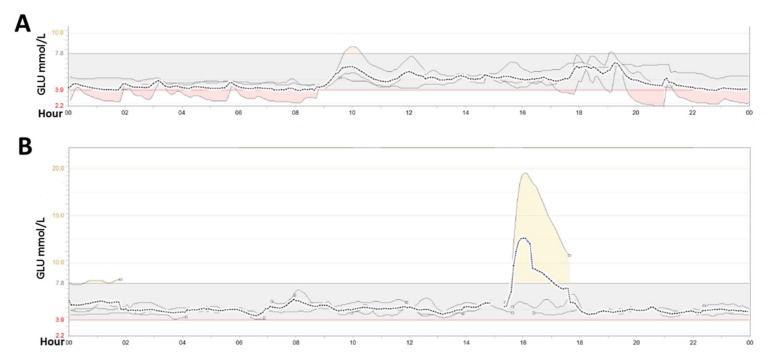
The dynamic blood glucose monitoring results. The blood glucose range was continuously detected for three days by an audiomonitor (Minimed Paradigm 722, Medtronic). (**A**) Hypoglycemia (<3.9 mmol/L) of Case 2 mainly occurred from 8:00 pm to 8:00 am; (**B**) Though no hypoglycemia was detected in Case 4, he did not appear to have a high postprandial blood glucose peak, which could be induced by oral glucose intake (the peak in 16:00–18:00). The dashed line represents the mean value of glucose over three days. The three solid line represents the results of each day monitoring result, respectively. The small circle after the solid line means monitoring interruptions because of non-cooperation of the patient. GLU: glucose.

Case 2 was a girl of seven years and three months old and who was taken to the neurology ward at SCMC due to convulsions. The patient was a full-term baby and her parents were physical healthy, with a non-consanguineous marriage. Before she was born, the couple had undergone two induced abortions. Her neonatal period and infancy were normal. At four years old, she fell ill with vomiting and developed lactic acidosis with hypoglycemia and convulsions, for which she was brought to the emergency department. She had a recurrence of these symptoms at five years and 10 months. There was no positive family history. The laboratory results showed hypoglycemia (glucose: 0.6 mmol/L), hyperlactacidemia (12.5 mmol/L), metabolic acidosis (pH: 6.88, base excess: −23.1 mmol/L), and hyperuricemia (uric acid: 599 μmol/L). After glucose infusion, results of blood samples showed insulin (23.8 μIU/mL), ACTH (9.13 pg/mL), and cortisol (8.9 μg/dL). The cranial MRI results showed her to have mildly enlarged left ventricle (Figure 1C,D). An ultrasound showed no abnormalities of the abdomen, thyroid, or heart. After treatment with an infusion of glucose to correct hypoglycemia, the patient was transferred to Department of Endocrinology for further therapy and investigation, where her dynamic blood was tested by glucose a continuous glucose monitoring system (CGMS, Medtronicn Minimed) for three days. The mean glucose level was 5.0 ± 0.8 mmol/L, the average area under the curve (AUC, <3.9 mmol/L) was 0.17, and hypoglycemia mostly occurred at night and in the morning (Figure 2A).

Case 3 was a girl of about six years and four months old, and she presented to the endocrinology clinic at SCMC because of recurrent hypoglycemia attacks. She was born as healthy full-term baby after a normal pregnancy and delivery, her elder sister was healthy, as well. There were no deaths or miscarriages reported by parents. The girl’s medical history revealed several previous hospitalizations, at the ages of eight months, three years, five years, and six years, due to vomiting, convulsions, fainting, and marked hypoglycemia. When hospitalized for a severe attacks at three years old, her laboratory results showed hypoglycemia (glucose: 2.0 mmol/L), metabolic acidosis (pH: 7.11, base excess: −25.6 mmol/L), hyperkalemia (7.3 mmol/L), hyperlactacidemia (10.4 mmol/L), hyperuricemia (uric acid 1218 μmol/L), and kidney dysfunction (creatinine: 143 μmol/L, urea nitrogen: 23.9 mmol/L), as well as liver damage (ALT: 84 U/L, AST: 118 U/L). After hypoglycemia was corrected, the level of insulin was 22.9 μIU/mL, ACTH was 18.86 pg/mL, and cortisol was 17.76 μg/dL. The cranial MRI and ultrasound for abdomen, thyroid, and heart appeared normal. At five years old, she was set up with a CGMS for three days in a local hospital, which demonstrated a stable glucose level (3.8–8.9 mmol/L), a mean glucose level of 5.9 mmol/L, and no obvious episodes of hypoglycemia were identified (no images).

Case 4 was a boy of 10 years and eight months old was referred to the department of SCMC’s Pediatric Intensive Care Unit for severe vomiting, convulsions, and coma. He was born full term by normal delivery, the third child of healthy parents. The first baby was premature and died of asphyxia at two months, whereas the second lived to 24 years old, and he was healthy. The first onset was 24 h after birth due to very poor oral intake. Due to frequent convulsions, he was diagnosed with epilepsy at the age of one year and received antiepileptic therapy. The attacks then tended to be less severe, and the drug treatment was terminated when he was three years old. However, through to age 10, his attacks of convulsions occurred frequently. He recently developed strabismus and vision loss involving the right eye (Figure 1H). The boy also had an obvious intellectual development delay. The initial laboratory findings revealed hypoglycemia (glucose: 0.6 mmol/L), hyperlactacidemia (12.6 mmol/L), metabolic acidosis (pH: 6.91, base excess: −22.1 mmol/L), and hyperuricemia (uric acid: 892 μmol/L). Blood samples obtained during hypoglycemia were insulin: 14 μIU/mL, ACTH; 19.6 pg/mL, and cortisol: 38.5 μg/dL. The cranial MRI results showed that several parts of the brain harbored symmetrical, patchy, abnormal signals, including the cerebellum, frontoparietal area, basal ganglia, and thalamus (Figure 1E,F). Cranial magnetic resonance spectroscopy (MRS) identified a significantly increased lactate peak with a reduced *N*-acetyl aspartate (NAA) peak (Figure 1G), indicating hypoxic lesions of the brain. Abdominal ultrasound showed hepatomegaly and pyoperitoneum. The CGMS data showed that no hypoglycemia occurred. The mean glucose level was 5.0 ± 0.5 mmol/L, yet postprandial glucose peaks were poor, and this was successfully induced by glucose (Figure 2B).

All of the clinical and laboratory data in the four cases are presented in Table 1.

### 2.2. Identification of the Mutations in FBP1 Gene

All four patients who had hypoglycemia, hyperlactacidemia, metabolic acidosis, and hyperuricemia, were screened for causal variants using targeted-NGS. The sequencing quality metrics information was listed in Figure S2 and Table S1 (see Supplementary Material). The screening parameters of the Ingenuity software of the candidate variants includes four steps: (1) exclusion of low confidence variants; (2) screening with criterion of an allele frequency under 1% in the 1000 Genomes Project or 3.0% of the National Heart, Lung, and Blood Institute Exome Sequencing Project Variant Server, or 3.0% of in the ExAC database, and the area of analysis area includes each exon and about 20 bp of exon-intron boundaries; (3) exclusion of benign variants; including synonymous, harmless missense predicted by PolyPhen-2 and SIFT software, and those predicted to have no impact on splicing by MaxEntScan software; and (4) clinical symptoms of hypoglycemia, hyperlactacidemia, metabolic acidosis, and hyperuricemia were chosen as the filtering index to analyze the above-screened candidate variants. Ultimately, variations in *FBP1* gene were found to contribute to the patients’ conditions.

**Figure 3:**
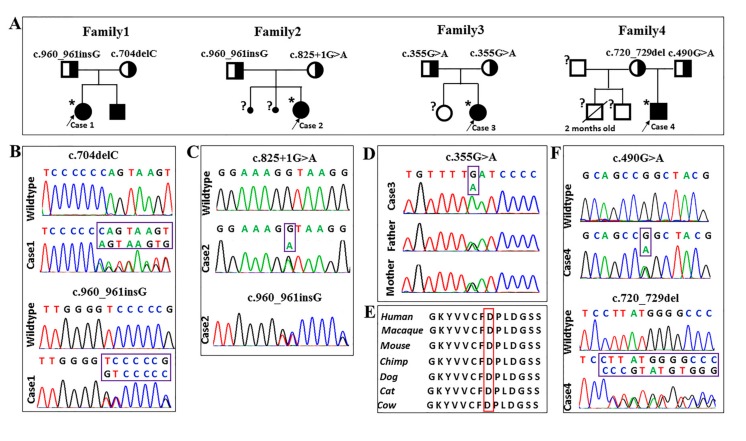
Pedigrees of the families and genetic sequencing findings. (**A**) The pedigrees of all investigated cases. Asterisks (*) indicate individuals subjected to next-generation sequencing, individuals marked with a question mark (?) were not genotyped for the *FBP1* variants. (**B**–**D**,**F**) Variants in *FBP1* gene identified by NGS were verified by Sanger sequencing. Three small deletions/duplications (c.704delC, c.960_961insG, and c.720_729del), two missense (c.355G>A and c.490G>A), and one splicing (c.825+1G>A) mutation were identified in the four patients, and all of the patients inherited the mutations from their parents, respectively. (**E**) The codon 119 of aspartate is highly-conserved in multiple species.

Compound heterozygous mutations were identified in Case 1 and her affected brother. One mutation was a novel deletion of a single base “cytosine” (c.704delC) in exon 5, which resulted in a frameshift leading to a premature stop codon (p.Pro235GlnfsX42). The other was a hot-spot mutant site (c.960_961insG) in exon 7, leading to a premature stop codon (p.Ser321ValfsX13) [10]. Direct sequencing results confirmed the results and revealed that the patient’s father was heterozygous for the c.704delC mutation, and her mother was heterozygous for the c.960_961insG mutation, which means the genetic mutations in this pediatric patient were inherited from his parents (Figure 3A,B). The NGS analysis results revealed Case 2 to have compound heterozygous mutations in the *FBP1* gene. The patient also had the mutation c.960_961insG. She also had a transversion of guanine to adenine (c.825+1G>A) in the first base of the 5′-splice site of intron 6. Direct sequencing results suggested that her father carried the mutation of c.960_961insG, while her mother carried the splice mutation of c.825+1G>A (Figure 3A,C). Case 3 had a homozygous missense mutation at codon 119 of exon 3 (c.355G>A), which led to an amino acid conversion (Asp119Asn), which occurred in a highly-conserved region. The mutation was also found in a heterozygous state in both of her parents (Figure 3A,D,E). For Case 4, a previously-identified c.490G>A (Gly164Ser) mutation in exon 4 [11] and a novel mutation of c.720_729del in exon 6 were identified. Both were heterozygous and inherited from his parents, respectively (Figure 3A,F). Sequence analysis showed the mutation of c.720_729del resulted in a premature stop codon (p.Tyr241GlyfsX33).

### 2.3. Down-Regulation of the FBPase Protein Expression and Enzymatic Activity by Mutant FBP1 Plasmids

**Figure 4:**
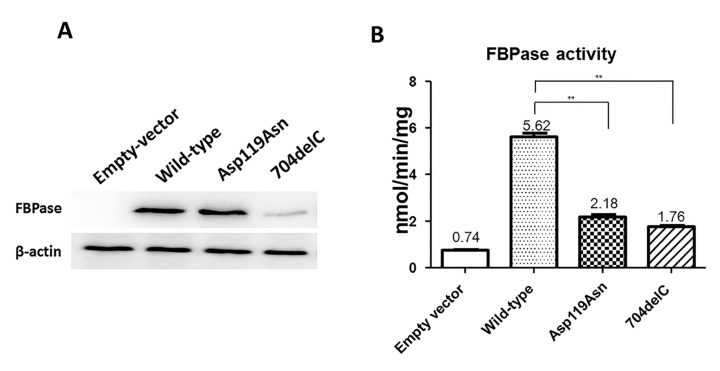
In vitro function studies of Asp119Asn and 704delC in COS-7 cell line. (**A**) The Western blotting results showed 704delC leads to a lower protein expression level as well as a slightly smaller protein size, Asp119Asn has no effect on the protein expression; (**B**) The FBPase activity of Asp119Asn and 704delC markedly decreased compared with that of the wild-type. The data represent mean values of FBPase activities performed three times. **: *p* value < 0.01

To determine whether the mutation of ASP119ASN affect FBPase protein functions, the eukaryotic expression plasmids of wild-type and the mutant *FBP1* were constructed and transiently transfected into COS-7 cells, 704delC was selected as a positive control because it could lead to a premature stop codon. Compared with the wild-type, the mutation of the 704delC not only caused a low protein expression level but also a slightly smaller sized protein, while the mutation of Asp119Asn had no impact on the protein expression (Figure 4A). The average enzymatic activity of the wild-type, Asp119Asn, and 704delC was 5.62, 2.18, and 1.76 nmol/min/mg protein, respectively (Figure 4B), indicating that the Asp119Asn largely reduces the FBPase activity.

### 2.4. Treatment and Follow-up

With molecular diagnosis confirmed, the use of fructose-free food and avoidance of prolonged fasting are recommended in these four patients with FBPase deficiency. In addition, in order to prevent hypoglycemia and metabolic acidosis, uncooked cornstarch (2 g/kg) had been used in these patients. During follow-up, there were no similar signs or symptoms.

## 3. Discussion

FBPase deficiency is a very rare inherited disease, first described by Baker and Winegrad in 1970 [12]. The incidence is estimated to be between 1/350,000 and 1/900,000 [9,13], but no data is available in the Chinese population. The disorder is characterized by recurrent episodes of hypoglycemia and metabolic acidosis during fasting, with symptoms usually manifesting during the first days of life [12]. Laboratory tests usually uncover marked hypoglycemia, lactic acidosis, and elevated levels of uric acid, while liver and kidney functions in most patients have always been reported to be normal [9]. Hypoglycemia-induced insulin drops in insulin and counter-regulatory hormones increase (ACTH, cortisol, and growth hormone) [14]. The attacks decrease with age, and most cases exhibit normal growth and development. If the treatment for hypoglycemia is properly administered, the prognosis is good [9,12].

This study describes four patients who presented acute metabolic crisis with the identical symptoms of unexplained hypoglycemia, acidosis, and convulsions in their medical history. In Case 4, who experienced the most severe attacks, the first onset was 24 h after birth. Due to frequent convulsions and cerebral anoxia, there was obvious organic damage to his brain, which has affected his intellectual development and vision. Curiously, his bilateral brain damage was unable to explain the blindness in his right eye. Hypoglycemia leading to convulsions in the patients can be relieved by glucose infusion, and long-term hypoglycemia may cause hypoglycemic encephalopathy, thereby showing symptoms of epilepsy, although Case 4 was diagnosed with epilepsy at the age of one year, indicating the heterogeneity of clinical features in FBPase-deficient patients. Dynamic blood glucose monitoring was performed on Cases 2 and 4 to determine when hypoglycemia would occur to guide the parents to arrange for reasonable daily diet. FBPase deficiency is often misdiagnosed as several other metabolic disorders, such as G6P deficiency and PGM1 deficiency. Both have contributed to the dearth of knowledge and difficulty of diagnosis for pediatricians. Though three patients had their first onset before the age of two years, because it was not possible to make a clear diagnosis, the families did not understand how to prevent the next event, leaving the patients’ lives to be threatened once again. They were only able to treat the symptoms via glucose supplementation, anti-infection treatments, and acidosis correction. The earlier the cause of blood glucose irregularities is determined and the sooner the blood glucose level is controlled, the better the prognosis for the FBPase-deficient patients, indicating the importance of suitable diagnostic methods.

**Figure 5:**
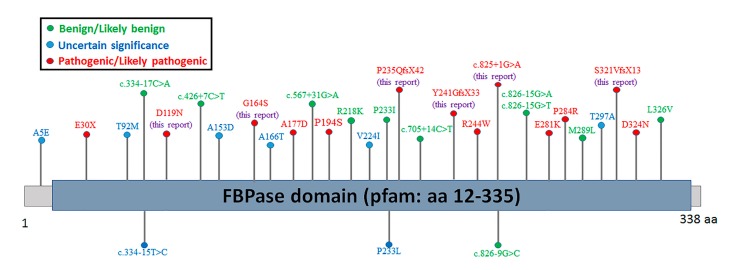
Distribution schematic of the variants of *FBP1* gene in the ClinVar database. aa: amino acid.

Since the first mutation was reported in 1995 [10], 35 different mutations have been reported, including 12 missense mutations, 12 deletion mutations, four nonsense mutations, four insertions/duplications, two splices, and one indel [13]. It has already been shown that next-generation sequencing is particularly suitable for the molecular diagnosis of complex metabolic disease [15]. Here, all four patients were definitively diagnosed with FBPase deficiency using next-generation sequencing. Genetic sequencing analysis of *FBP1* gene showed Case 1 to have a compound heterozygous mutation involving c.704delC and c.960_961insG. c.960_961insG was found to be the most common causative mutant site described in patients from Asia, Europe, and North America [9,10,16], which was referenced in the ClinVar database as pathogenic (https://www.ncbi.nlm.nih.gov/clinvar/variation/867/). It is also the only one reported in China [17]. The 704delC is novel. Case 2 involved a compound heterozygote for c.960_961insG and c.825+1G>A, and the splicing mutation had been reported in a French patient [9]. Case 3 was homozygous for Asp119Asn, though it was not a novel variation (allele frequency in ExAC database is 0.00165%), and was classified as likely pathogenic in the ClinVar database (https://www.ncbi.nlm.nih.gov/clinvar/variation/214364/), and no functional studies have been assessed. Case 4 showed the compound heterozygote of Gly164Ser (pathogenic in the ClinVar database, https://www.ncbi.nlm.nih.gov/clinvar/variation/868/) and 720_729del, the functions of Gly164Ser have been thoroughly studied in previous works [11,18], but 720_729del is novel. Additionally, two compound mutations (Gly164Ser and 960_961insG, Gly164Ser and 838delT) have been characterized [11,18], but the compound of Gly164Ser and 720_729del is novel. According to the standards issued by the American College of Medical Genetics and Genomics [19], the novel mutations of 704delC and 720_729del are pathogenic (PVS1 + PM2 + PP4). Functional studies have demonstrated the missense mutation of Asp119Asn can affect the enzyme activity, with which Asp119Asn has been determined to be likely pathogenic (PS3 + PM2 + PP3 + PP4+ PP5). What was unexpected is that the 704delC does not lead to a null protein, indicating the truncated protein did not induce the nonsense-mediated mRNA decay (NMD). Previous studies have shown that 838delT and 960dupG lead to null proteins and total loss of enzyme activity [10,18]. The mechanism by which 704delC fails to induce NMD need further investigation. Interestingly, all of the six variants in this study combined with the pathogenic/likely pathogenic variants of *FBP1* gene from the ClinVar database are located in the pfam domain (Figure 5). Additionally, in order to better understand the six identified variants, an annotation table was listed (Table 2).

There was no association between genetic mutations and clinical manifestations in patients with FBPase deficiency. The patients presented here have different forms of the disease, despite sharing the same mutation: age of onset was very different for Case 1 and her brother (72 h for Case 1 and five months for her brother), suggesting that the genotype cannot predict the age of onset of the disease.

In summary, four Chinese patients with FBPase deficiency were described and diagnosed using targeted-next generation sequencing. Two novel frame shift mutations, c.704delC (p.Pro235GlnfsX42) and c.720_729del (p.Tyr241GlyfsX33), were identified. Additionally, the in vitro function studies demonstrated the missense mutation (c.355G > A, p.Asp119Asn) and c.704delC, indeed, changed the protein expression or the enzyme activities. This report not only expands upon the spectrum of variation of *FBP1* gene, but also deepens our understanding of the clinical features associated with FBPase deficiency.

## 4. Materials and Methods

### 4.1. Approval of the Institutional Review Board and Informed Consent

All procedures followed were in accordance with the ethical standards of the responsible institutional committee on human experimentation and with the Helsinki Declaration of 1975, as revised in 2000, and the protocol was approved by the Ethics Committee of Shanghai Children’s Medical Center (SCMCIRB-K2016013, 18 February 2016). Informed consent was obtained from each patient’s family.

### 4.2. Targeted Next Generation Sequencing and Data Analysis

The genomic DNA of the patients was isolated from 2-mL peripheral blood samples collected from the cubital veins using a QIAamp Blood DNA Mini Kit^®^ (Qiagen GmbH, Hilden, Germany). A total of 3 μg DNA from the patients was processed through shearing using a Covarias^®^ M220 Ultrasonicator system (Covaris, Inc., Woburn, MA, USA) to pieces 150–200 bp in size. An adapter-ligated library was produced with Agilent SureSelect Target Enrichment System (Agilent Technologies, Inc., Santa Clara, CA, USA) in accordance with the manufacturer’s instructions. The capture library was performed using an XT Inherited Disease Panel (cat No. 5190–7519, Agilent Technologies, Inc., Santa Clara, CA, USA), containing 2742 genes. The quality and size range of prepared library and the quantity of each index-tagged library were evaluated by using Agilent 2200 Bioanalyzer with high sensitivity DNA kit and Q-PCR NGS Library Quantification Kit (Agilent Technologies, Inc., Santa Clara, CA, USA), as well as Qubit^®^ 2.0 Fluorometer (Invitrogen, Thermo Fisher, Waltham, MA, USA). Clusters were then generated by isothermal bridge amplification using an Illumina cBot station, and sequencing was performed on an Illumina HiSeq 2500 System (Illumina, Inc., San Diego, CA, USA).

Base calling and sequence read quality assessments were performed using Illumina HCS 2.2.58 software (Illumina) for the Illumina HiSeq 2000 system, which included new versions of HiSeq control software and real-time analysis. Alignment of the sequence reads to a reference human genome (Human 37.3; SNP135) was performed using NextGENe^®^ (SoftGenetics LLC, State College, PA, USA). All single nucleotide variants (SNVs) and indels were saved in a VCF format file, and uploaded for Ingenuity^®^ Variant Analysis™ (Ingenuity Systems, Mountain View, CA, USA) for biological analysis and interpretation.

### 4.3. Sanger Sequencing Verification of the FBP1 Gene

The primers for amplification of the *FBP1* gene (GenBank accession no NM_000507.3) were designed using UCSC ExonPrimer online software (http://genome.ucsc.edu/index.html) and synthesized by Map Biotechnology (Shanghai, China). The primers designed for exons 3–7 are listed in Table S2 and the in silico analysis results of the primers are showed in Figure S3 (see Supplementary Material). The exons and the exon-intron boundaries were amplified using polymerase chain reaction (PCR). The PCR products (5 μL) were examined on a 1% agarose gel and purified using ExoSAP-IT Kit (GE Healthcare, Cleveland, OH, USA). Sequencing reactions were prepared with the BigDye^®^ Direct Cycle Sequencing Kit (Life Technologies, Thermo Fisher, Waltham, MA, USA). The final products were purified from agarose gel using QIAquick Gel Extraction Kit (Qiagen, Hilden, Germany) and the capillary electrophoresis sequencing was performed by using an ABI Prism 3730XL Genetic Analyzer (Applied Biosystems; Thermo Fisher, Waltham, MA, USA) with the forward and reverse primers. The sequence data were analyzed using Mutation Surveyor^®^ software version 4.0.4 (SoftGenetics, State College, PA, USA).

### 4.4. Plasmid Construction

The open reading frame (ORF) sequences of wild-type, Asp119Asn-mutant, and 704delC-mutant *FBP1* were synthesized by Shanghai Sangon (Shanghai, China), and the synthetic DNA were confirmed by Sanger sequencing. The restriction enzyme sites HindIII and PSTI were introduced into the DNA sequences to enable directional cloning. Target fragments were ligated into the pGEFP-N1 vector (Takara, Dalian, China) using solution I ligase (Takara, Dalian, China), in accordance with the manufacturer’s protocol.

### 4.5. Cell Culture and Cell Transfection

COS-7 cells were grown in Dulbecco’s modifid Eagle's medium supplemented with 10% (*v*/*v*) fetal bovine serum (Gibco; Thermo Fisher, Waltham, MA, USA) and 1% penicillin/streptomycin (Gibco; Thermo Fisher, Waltham, MA, USA) in a 5% CO_2_ incubator at 37 °C. Cell transfection was performed using Lipofectamine 2000 (Invitrogen; Thermo Fisher, Waltham, MA, USA), in accordance with the manufacturer’s protocol.

### 4.6. Western Blotting and Antibodies

To prepare whole-cell extracts, cells were washed once with ice-cold PBS and lysed with lysis buffer (100 mM Tris-cl, pH 6.8, 4% sodium dodecyl sulfonate, 20% glycerol, 0.1 mM phenylmethanesulfonyl fluoride). Equal amounts of samples were subjected to SDS–PAGE. After separation in the gel, the protein was transferred on a nitrocellulose membrane. Membranes were blocked in 2% BSA in TBS-T for 1 h, and then incubated overnight (4 °C) with antibodies against FBP1 (1:1000 Abcam, ab109732) and anti-β-actin (1:3000 Abcam, ab8226). After being washed in TBS-T, membranes were incubated with IRDye^®^ 800CW goat anti-mouse IgG secondary antibody (1:5000; LI-COR Biosciences, Inc., Lincoln, NE, USA) for 0.5 h at room temperature The FBP1 and β-actin were detected using a two-color infrared fluorescence imaging system (Odyssey; LI-COR Biosciences, Inc., Lincoln, NE, USA).

### 4.7. FBPase Activity Assays

COS-7 cells were cultured in the 60-mm-diameter dish, when the cells were 80% to 90% confluent, they were transfected with 5 μg wild-type or mutant plasmids, respectively. 48 h after transfection, the FBPase activity was measured using a kit (cat No. FDP-1-G) purchased from Comin Biotechnology (Suzhou, China, http://www.cominbio.com). The FBPase activity was measured using NADPH coupled spectrophotometric assay, in accordance with the manufacturer’s protocol.

### 4.8. Statistical Analysis

The statistical analysis was performed by Student’s *t*-test using the SPSS 19.0 software (SPSS 19.0, IBM, Armonk, NY, USA). A *p*-value of less than 0.05 was considered significant in all cases (* *p* < 0.05; ** *p* < 0.01; *** *p* < 0.001).

## Figures and Tables

**Table 1:** Summary of clinical findings of the patients.

Patients	Case 1	Case 2	Case 3	Case 4
General information				
Sex	Female	Female	Female	Male
Age at onset	72 h	4 years	8 months	24 h
Age at diagnosis	5 years-6 months	7 years-3 months	6 years-4 months	10 years-8 months
Birth height (cm)/weight (kg)	50/3.2	unknown/3.6	unknown/unknown	unknown/3.9
Current height (cm)/weight (kg)/BMI (kg/m^2^)	128 (3.37 SD)/25 (2.68 SD)/15.2	130 (1.15 SD)/23 (−0.11 SD)/13.6	123 (−0.01 SD)/27 (2.58 SD)/17.8	145 (0.22 SD)/35 (−0.51 SD)/16.6
**Results of blood chemistry**				
RBC (3.70–5.80 × 10^12^/L)	4.51	4.16	4.13	4.15
Hemoglobin (110–160 g/L)	128	115.0	117.0	119.0
Glucose (3.9–6.0 mmol/L)	0.3	0.6	2.0	0.6
pH of arterial blood (7.35–7.45)	6.97	6.88	7.11	6.91
Base excess (−3~+3 mmol/L)	−25.0	−23.1	−25.6	−22.1
Lactic acid (0.7–2.1 mmol/L)	11.9	12.5	10.4	12.6
Insulin (1.9–23.0 μIU/mL)	8.0	23.8	22.9	14.0
C-peptide (1.1–4.4 ng/mL)	3.06	2.08	3.11	2.89
Cortisol (5.70–16.60 ug/dL)	57.30	8.90	17.70	38.50
ACTH (8.00–80.00 pg/mL)	>1250.00	9.13	18.86	19.60
Sodium (135–145 mmol/L)	136	142	131	136
Potassium(3.5–5.0 mmol/L)	5.6	5.2	7.3	5.2
Chloride (98–110 mmol/L)	99	106	106	109
Uric acid (146–369 μmol/L)	875	599	1218	892
Creatinine (44–97 μmol/L)	71	52	143	64
Urea nitrogen (2.5–6.1 mmol/L)	6.4	7.5	23.9	7.8
ALT (0–40 U/L)	18	10	84	22
AST (0–45 U/L)	20	27	118	48
**Others findings**				
Ultrasound (thyroid, abdomen, and heart)	Normal	Normal	Normal	Hepatomegaly and pyoperitoneum
Electroencephalogram	Normal	Normal	Normal	Normal
Brain MRI	Enlarged bilateral ventricles	Enlarged left ventricle	Normal (no images)	Patchy abnormal signals
Intellectual development	Normal	Normal	Normal	Delayed

SD: standard deviation; BMI: body mass index; RBC: red blood cell; ALT: alanine aminotransferase; AST: aspartate aminotransferase; MRI: magnetic resonance imaging; ACTH: adrenocorticotropic hormone.

**Table 2:** Annotations of the identified variations in *FBP1* gene.

Patient	Genomic Position (NG_008174.1)	Transcriptional Position (NM_000507.3)	Amino Acid Position (NP_000498.2)	Exon/Intron Position (NM_000507.3)	Mutation Type	Homo/Het	Read Depth	ClinVar ID	CADD Score	Novelty	Carrier
Case 1	g.38434delC	c.704delC	p.Pro235GlnfsX42	Exon 5	Frameshift	Het	13	NA	NA	Novel	Father
	g.41812_41813insG	c.960_961insG	p.Ser321ValfsX13	Exon 7	Frameshift	Het	43	867	NA	Known	Mother
Case 2	g.39794G>A	c.825+1G>A	NA	Intron 6	Splicing	Het	108	NA	NA	Known	Mother
		c.960_961insG				Het	65				Father
Case 3	g.27411G>A	c.355G>A	p.Asp119Asn	Exon 3	Missense	Homo	167	214,364	33.000	Known	Father/Mother
Case 4	g.35252G>A	c.490G>A	p.Gly164Ser	Exon 4	Missense	Het	187	868	29.900	Known	Father
	g.39688_39697del	c.720_729del	p.Tyr241GlyfsX33	Exon 6	Frameshift	Het	93	NA	NA	Novel	Mother

NA, not available; Homo, homozygote; Het, heterozygote.

## Supplementary Materials

Supplementary materials can be found at www.mdpi.com/1422-0067/18/4/857/s1.
