# Supplementary material for: Clinical and Molecular Characterization of Patients with Fructose 1,6-Bisphosphatase Deficiency
Source: Int J Mol Sci. 2017 Apr 18;18(4):857. doi: 10.3390/ijms18040857 (PMC5412439; doi:10.3390/ijms18040857)
Supplement: Supplementary file 1 [file ijms-18-00857-s001.pdf]

Table S1. The sequencing quality metrics of four cases included in this study.

Patient	Total Reads	Average read depth (×)	Percent of ROI with 20× (%)	Percent of ROI with 1× (%)	Unaligned reads	Off target percent of reads (%)	Total SNVs	Non-synonymous SNVs
Case 1	113,198,302	146	96.4	99.9	565,990	35.5%	6456	5698
Case 2	107,309,066	140	95.7	99.8	493,621	36.4%	5287	3778
Case 3	124,999,609	163	96.8	99.8	999,998	33.0%	5605	4050
Case 4	114,539,275	152	96.3	99.9	801,775	34.7%	5362	3827

ROI: region of interest

Table S1. (Continued).

SNPs of SNVs	Total Indels	Prioritized by Ingenuity
5387	282	105
4576	161	97
4726	201	108
4596	180	94

Table S2. Primers used in amplifying exons and the flanking sequences of *FBP1*.

Exon	Forward Primer(5′-3′)	Reverse Primer(5′-3′)	Product Size (bp)
3	TGACAGTACAAATAAATAAGACCCAC	TGAGATACAAATCTACTTCAGTCTCAC	358
4	GGAAGGCTGCCGTGATAATC	CTCCACATACCCCTGCCAAT	408
5	GCTCGTCATCTCTCCACCTT	AGAACTTCATCGTGGGCTCA	418
6	GTGCTCGGTGAATGGATGTG	TCGTCCTGACCGTTCATTCT	464
7	CCACCAGCTCTGCATGTTTT	TTTGGTTAGGGAGTGCC	452

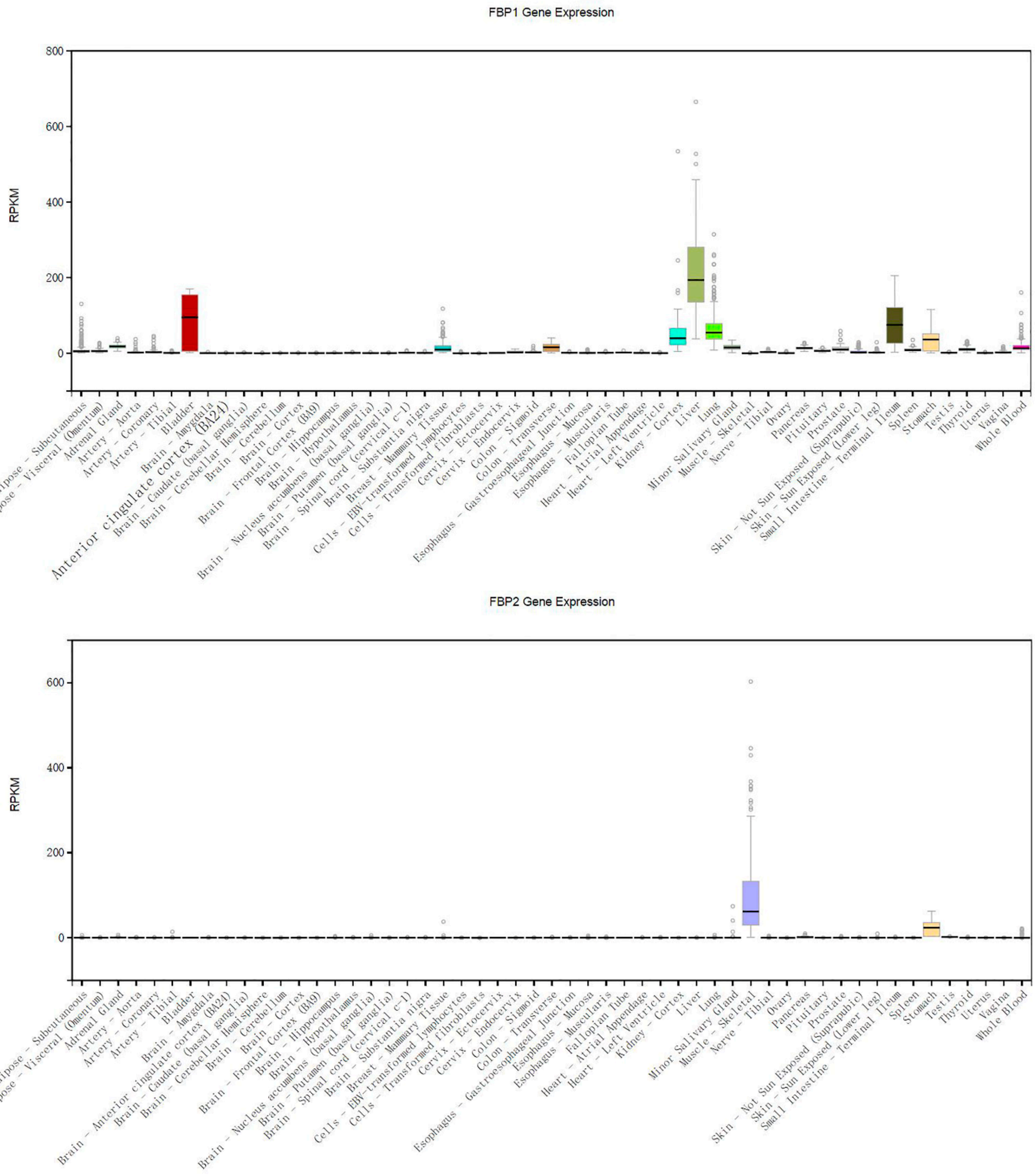

Figure S1. Expression pattern for FBP1 and FBP2 among the various tissues from GTEx Portal database. (A) The FBP1 protein mainly expresses in the liver, bladder, small intestine, kidney, lung, and stomach; (B) The FBP2 protein mainly expresses in the muscle and stomach.

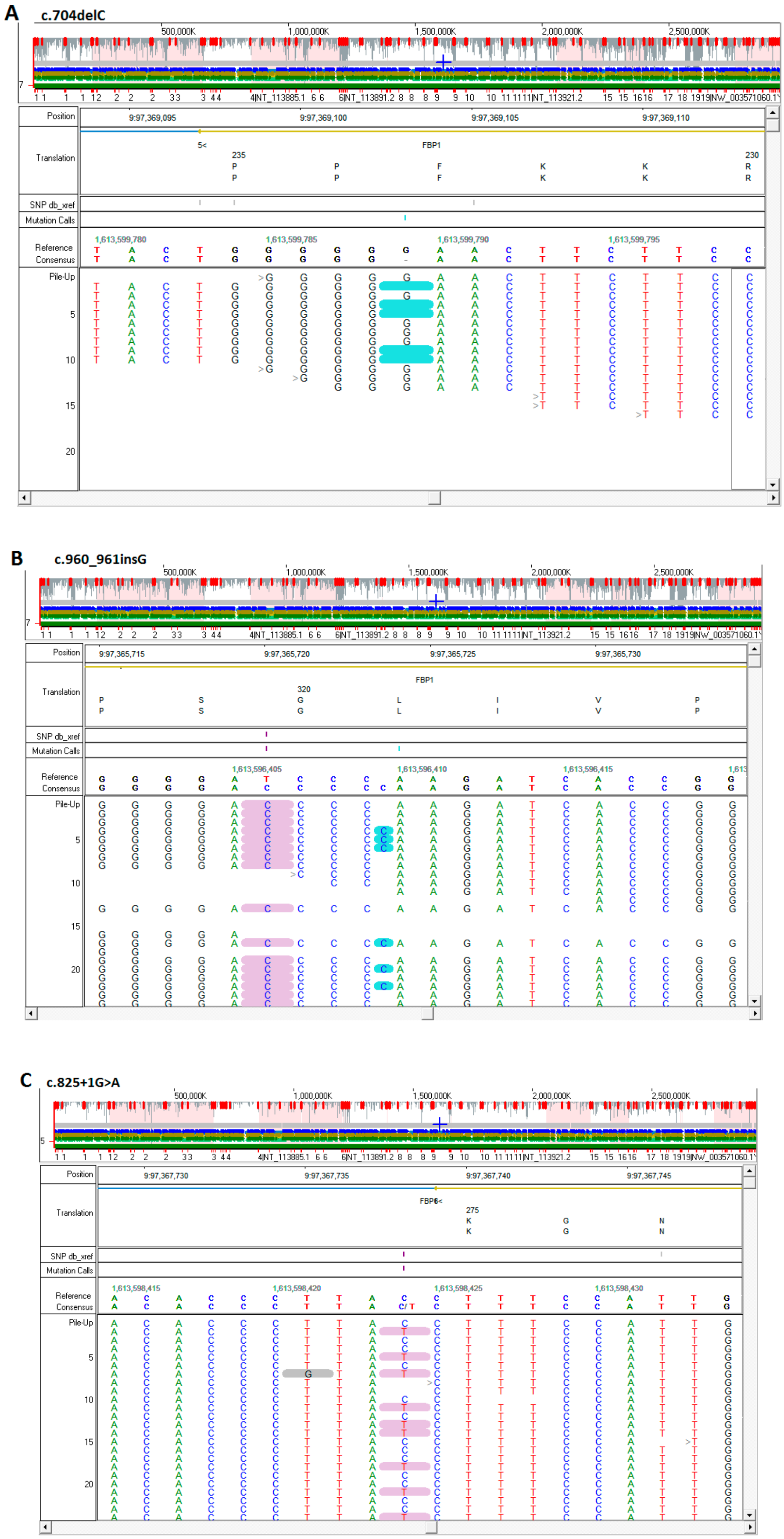
A
c.704delC
Position
Translation
FBP1
SNP db_xref
Mutation Calls
Reference
Consensus
Pile-Up
B
c.960_961insG
Position
Translation
FBP1
SNP db_xref
Mutation Calls
Reference
Consensus
Pile-Up
C
c.825+1G>A
Position
Translation
SNP db_xref
Mutation Calls
Reference
Consensus
Pile-Up

D **c.960_961insG**

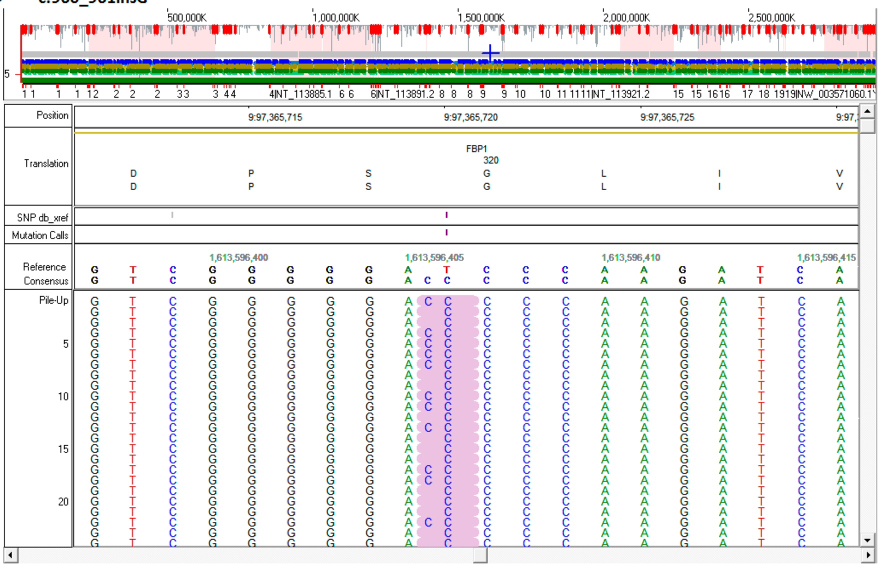

E **c.355G>A**

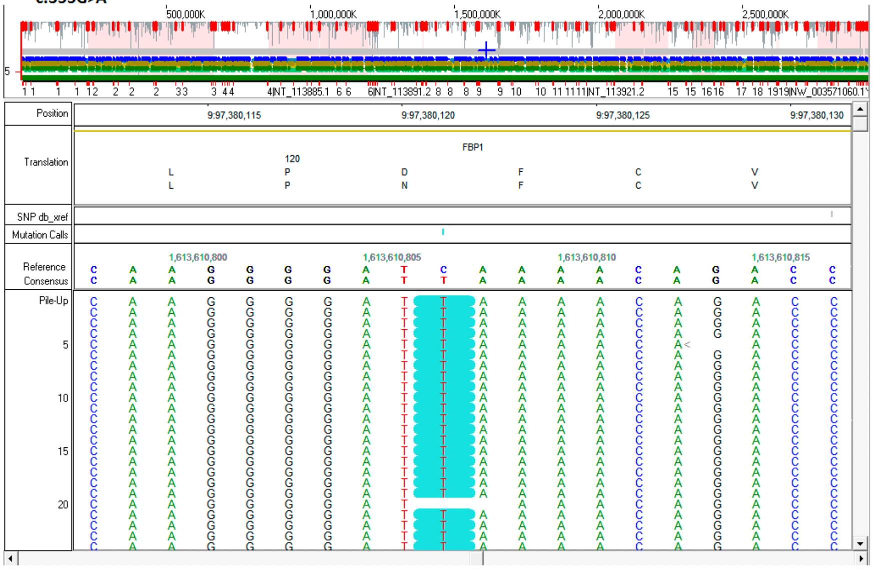

F **c.490G>A**

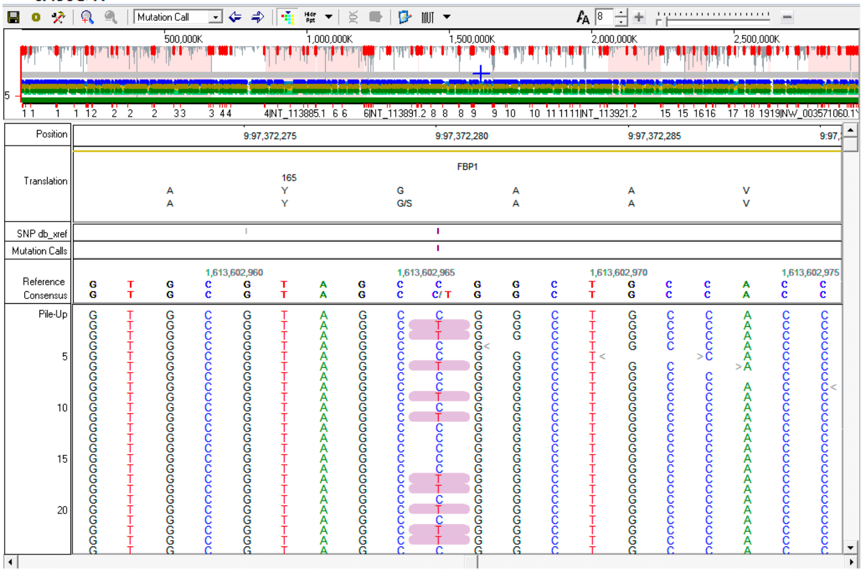

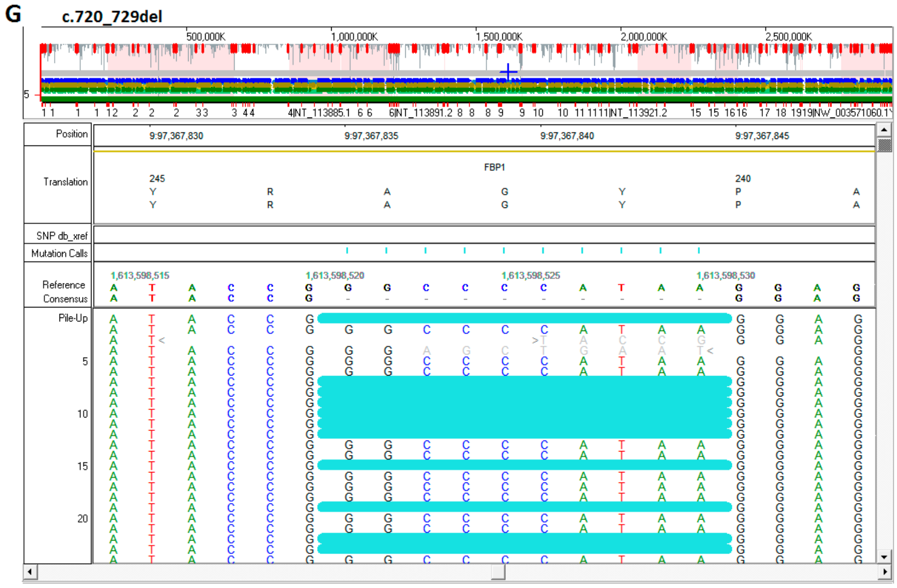

Figure S2. Reads alignment in the mutated positions for all the cases. Sequence alignment was performed by the NextGENe® software. (A, B), (C, D), (E), and (F, G) indicate the reads alignment in the mutated positions for case 1, 2, 3, and 4, respectively.

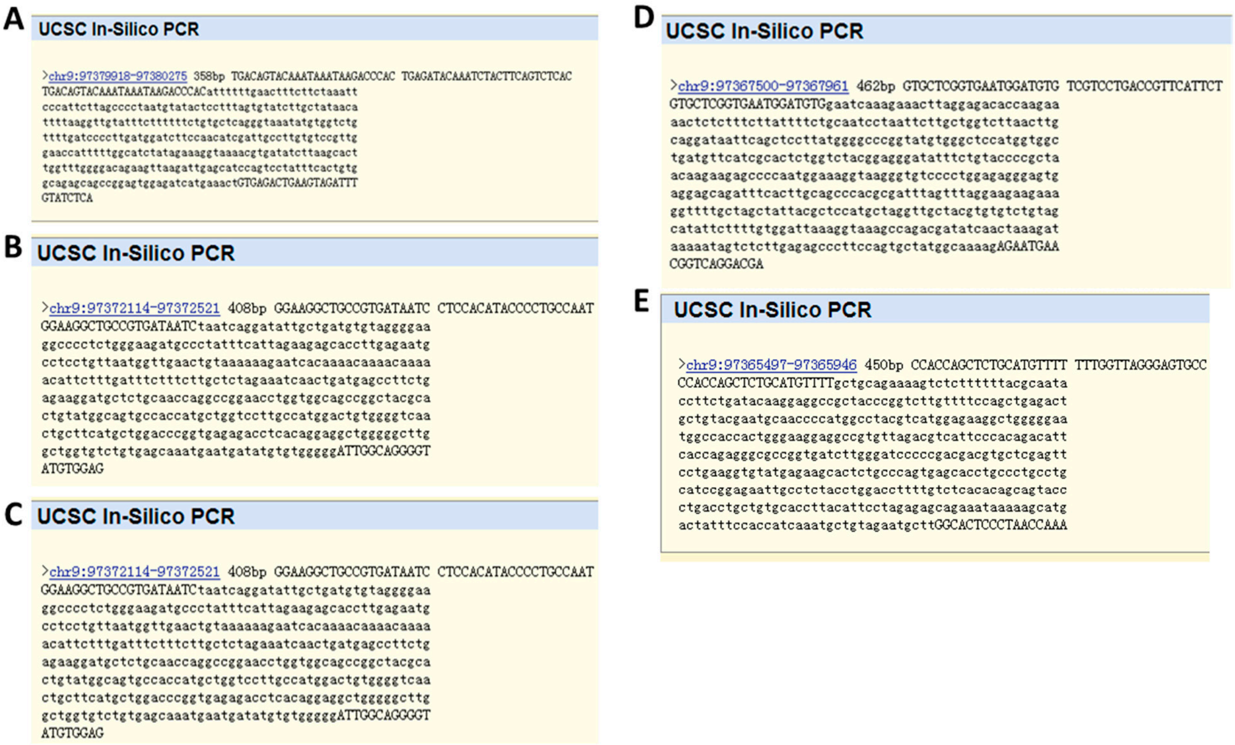

Figure S3. *In-silico* analysis of the primers. The effectiveness analysis of the five pair of primers to amplify the exon 3-7 of FBP1 gene were performed by UCSC (http://genome.ucsc.edu). The analysis results showed that all the primers are appropriate (A–E).
